# Association of the *APOA1* rs964184 SNP and serum lipid traits in the Chinese Maonan and Han populations

**DOI:** 10.1186/s12944-018-0759-8

**Published:** 2018-05-10

**Authors:** Ling Qiu, Rui-Xing Yin, Eksavang Khounphinith, Fen-Han Zhang, De-Zhai Yang, Shang-Ling Pan

**Affiliations:** 1 Department of Cardiology, Institute of Cardiovascular Diseases, The First Affiliated Hospital, Guangxi Medical University, 22 Shuangyong Road, Nanning, 530021 Guangxi People’s Republic of China; 2 Department of Molecular Genetics, Medical Scientific Research Center, Guangxi Medical University, Nanning, 530021 Guangxi People’s Republic of China; 3 Department of Pathophysiology, School of Premedical Sciences, Guangxi Medical University, Nanning, 530021 Guangxi People’s Republic of China

**Keywords:** Lipids, Apolipoprotein A1, Single nucleotide polymorphism, Environmental factors

## Abstract

**Background:**

Maonan nationality belongs to a mountain ethnic minority in China. Little is known about the association of apolipoprotein A1 gene (*APOA1*) rs964184 single nucleotide polymorphism (SNP) and serum lipid levels in this population. The aim of this study was to detect the association of the *APOA1* rs964184 SNP and several environmental factors with serum lipid profiles in the Chinese Maonan and Han populations.

**Methods:**

Genotypes of the *APOA1* rs964184 SNP in 867 individuals of Maonan nationality and 820 participants of Han nationality were determined by polymerase chain reaction and restriction fragment length polymorphism, combined with gel electrophoresis, and confirmed by direct sequencing.

**Results:**

The frequencies of CC, CG and GG genotypes of the *APOA1* rs964184 SNP were 68.86, 29.18 and 1.96% in the Maonan population, and 63.78, 30.85 and 5.37% in the Han population (*P* < 0.001). The frequency of the G allele was 16.55% in Maonan and 20.79% in Han (*P* < 0.001). The G allele carriers had lower high-density lipoprotein cholesterol (HDL-C) levels in Maonan and higher triglyceride (TG) levels in Han peoples than the G allele non-carriers. Subgroup analyses showed that the G allele carriers had lower HDL-C levels in both Maonan males and females; and lower apolipoprotein (Apo) A1 levels and the ApoA1/ApoB ratio in Han males than the G allele non-carriers. Serum lipid parameters in the two ethnic groups were also associated with several environmental factors.

**Conclusions:**

The present study reveals that there may be a racial/ethnic- and/or gender-specific association between the *APOA1* rs964184 SNP and serum lipid parameters in our study populations.

**Trial registration:**

Retrospectively registered.

## Background

Nowadays cardiovascular disease (CVD) becomes the primary cause of disability among adults found in some studies, and coronary artery disease (CAD) continues to be the leading cause of death and years of life lost around the world [1, 2]. The number of deaths from CAD has increased 35% over the 20 years from 1990 to 2010 [2]. It is widely acknowledged that dyslipidemia is one of the major risk factors for CVD from its prediction to development [3]. Dyslipidemia highly relates to the risk of CAD, which related to increased serum levels of total cholesterol (TC) [4], triglyceride (TG) [5], low-density lipoprotein cholesterol (LDL-C) [6], and apolipoprotein (Apo) B [7], combined with decreased levels of high-density lipoprotein cholesterol (HDL-C) [8], ApoA1 and the ApoA1/ApoB ratio [8]. Several previous studies have demonstrated that low level of HDL-C is an independent risk factor of CAD [9, 10].

It is well established that abnormal serum lipid levels are concerned with genetic and multiple environmental factors and their interactions [11]. ApoA1 gene (*APOA1*) encodes a protein, ApoA1, in humans, which has a specific role in lipid metabolism [12, 13]. There is another study showed that the pathway of HDL-C metabolism involves Apo, and ApoA1 is the most abundant component of HDL-C [14]. *APOA1* plays an essential role in formation, maturation and metabolism of HDL-C [15]. ApoA1 is also a better biomarker of cardiovascular risk [10]. Genome-wide association study (GWAS) has identified that genetic variant of the *APOA1* rs964184 SNP was associated with serum TG, TC, HDL-C and LDL-C levels [16]. However, it has not been verified that the effect of the *APOA1* rs964184 SNP on serum lipid levels, both in the mechanism and function. To the best of our knowledge, the association of the *APOA1* rs964184 SNP and serum lipid levels has not been previously reported in the Maonan population. Therefore, this study was undertaken to assess the association of the *APOA1* rs964184 SNP and several environmental factors with serum lipid profiles in the Maonan and Han populations.

## Methods

### Subjects

A total of 867 unrelated participants (329 males, 37.95% and 538 females, 62.5%) of Maonan nationality and 820 unrelated subjects (306 males, 37.32% and 514 females, 62.68%) of Han nationality were randomly selected from our previous stratified randomized samples [17]. The participants were all agricultural workers from Huanjiang Maonan Autonomous County, Guangxi Zhuang Autonomous Region, People’s Republic of China. These participants’ age ranged from 22 to 92 years with the mean age of 57.71 ± 14.74 years in Maonan and 56.34 ± 15.63 years in Han, respectively. The age distribution and gender ratio were matched between the two ethnic groups. All participants were essentially healthy with no evidence of CVD such as CAD, stroke, diabetes, hyper- or hypo-thyroids, and chronic renal disease. They were free from any treatment which would affect serum lipid levels. This study design was approved by the Ethics Committee of the First Affiliated Hospital, Guangxi Medical University (No. Lunshen-2014-KY-Guoji-001, Mar. 7, 2014). Informed consent was obtained from all participants before study.

### Epidemiological survey

The epidemiological survey was performed using internationally standardized methods, following a common protocol [18]. Information on demographics, socioeconomic status, and lifestyle factors was collected with standardized questionnaires. The intake of alcohol was quantified as the number of liang (about 50 g) of rice wine, corn wine, rum, beer, or liquor consumed during the preceding 12 months. Alcohol consumption was categorized into groups of grams of alcohol per day: 0 (non-drinker), ≤ 25 and > 25. Smoking status was categorized into groups of cigarettes per day: 0 (non-smoker), ≤ 20 and > 20. Several parameters such as height, weight, blood pressure, and waist circumference were measured. Body mass index (BMI) was calculated as weight/height^2^ (kg/m^2^).

### Biochemical measurements

A fasting venous blood sample of 5 ml was drawn from the participants. A part of the sample (2 mL) was collected into glass tubes and used to determine serum lipid levels. Another part of the sample (3 mL) was transferred to tubes with anticoagulants (4.80 g/L citric acid, 14.70 g/L glucose and 13.20 g/L tri-sodium citrate) and used to extract deoxyribonucleic acid (DNA). Measurements of serum TC, TG, HDL-C, and LDL-C levels in the samples were performed by enzymatic methods with commercially available kits (RANDOX Laboratories Ltd., Ardmore, Diamond Road, and Crumlin Co. Antrim, United Kingdom, BT29 4QY; Daiichi Pure Chemicals Co., Ltd., Tokyo, Japan). Serum ApoA1 and ApoB levels were detected by the immunoturbidimetric immunoassay using a commercial kit (RANDOX Laboratories Ltd.). All determinations were performed with an auto-analyzer (Type 7170A; Hitachi Ltd., Tokyo, Japan) in the Clinical Science Experiment Center of the First Affiliated Hospital, Guangxi Medical University [19].

### DNA amplification and genotyping

Genomic DNA of the samples was isolated from peripheral blood leucocytes according to the phenol-chloroform method [20]. The extracted DNA was stored at 4 °C until analysis. Genotyping of the *APOA1* rs964184 SNP was performed by polymerase chain reaction and restriction fragment length polymorphism (PCR-RFLP). PCR amplification was performed using 5′-CCATTTCCTTGCACAACCCA-3′ and 5′-ACTGGCCTCTGTATTGACCC-3′ as the forward and reversed primer pair, respectively. (Sangon, Shanghai, People’s Republic of China). Each amplification reaction was performed in a total volume of 25.0 μL, including 12.5 μL of 2 × *Taq* PCR MasterMix (constituent: 0.1 U *Taq* polymerase/μL, 500.0 μM dNTP each and PCR buffer), DNase/RNase-free water (ddH_2_O) 8.5 μL, 1.0 μL each primer (10 pmol/L) and 2.0 μL genomic DNA, processing started with 5 min of pre-denaturing at 95 °C and followed by 30 s of denaturing at 95 °C, 30 s of annealing at 59 °C and 40 s of elongation at 72 °C for 33 cycles. The amplification was completed by a final extension at 72 °C for 7 min. Following electrophoresis on a 2.0% agarose gel with 0.5 μg/mL ethidium bromide, the amplification products were visualized under ultraviolet light. The restriction enzyme reaction consisted of 5.0 μL amplified DNA, 8.8 μL ddH_2_O, 1.0 μL of 10 × buffer solution and 0.2 μL *Mbo*I restriction enzyme [New England Biolabs (Beijing) Ltd.] in a total volume of 15 μL, which digested at 37 °C for 40 min. After restriction enzyme digestion of the amplified DNA, genotypes were identified by electrophoresis on 2% ethidium bromide stained agarose gels and visualized under ultraviolet light. Nine samples (each genotype in three; respectively) detected by the PCR-RFLP were also confirmed by direct sequencing. The DNA sequences were analyzed using an ABI Prism 3100 (Applied Biosystems) in Shanghai Sangon Biological Engineering Technology & Services Co., Ltd., People’s Republic of China.

### Diagnostic criteria

The normal values of serum TC, TG, HDL-C, LDL-C, ApoA1, ApoB levels and the ApoA1/ApoB ratio in our Clinical Science Experiment Center were 3.10-5.17, 0.56-1.70, 1.16-1.42, 2.70-3.10 mmol/L, 1.20-1.60, 0.80-1.05 g/L and 1.00-2.50, respectively. The individuals with TC > 5.17 mmol/L and/or TG > 1.70 mmol/L were defined as hyperlipidemic [20, 21]. Hypertension was diagnosed according to the 1999 and 2003 criteria of the World Health Organization-International Society of Hypertension Guidelines for the management of hypertension [22]. The diagnostic criteria of overweight and obesity were according to the Cooperative Meta-Analysis Group of China Obesity Task Force. Normal weight, overweight and obesity were defined as a BMI < 24, 24-28 and > 28 kg/m^2^, respectively [23].

### Statistical analyses

The statistical analyses were performed with the statistical software package SPSS 21.0 (SPSS Inc., Chicago, Illinois). The quantitative variables were presented as mean ± standard deviation (serum TG levels were presented as medians and interquartile ranges). Allele frequency was determined via direct counting, and the Hardy-Weinberg equilibrium was verified with the standard goodness-of-fit test. The genotype distribution between the two groups was analyzed by the chi-square test. General characteristics between two ethnic groups were compared by the Student’s unpaired *t*-test. The association between genotypes and serum lipid parameters was tested by covariance analysis (ANCOVA) with gender, age, BMI, blood pressure, alcohol consumption and cigarette smoking as covariates. Multivariable linear regression analyses with stepwise modeling were used to determine the correlation between the genotypes (CC = 1, CG = 2 and GG = 3) and several environmental factors with serum lipid levels in males and females of Maonan and Han populations. Two-sided *P* value < 0.05 was considered statistically significant.

## Results

### General characteristics and serum lipid profiles

The general characteristics and serum lipid levels between the Maonan and Han populations are summarized in Table 1. Systolic blood pressure, pulse pressure, serum TG, and ApoA1 levels were higher in Maonan than in Han (*P* < 0.05-0.001), whereas the percentages of cigarette smoking, the levels of blood glucose, TG and HDL-C were lower in Maonan than in Han (*P* < 0.001). There was no significant difference in the gender ratio, age structure, body height, weight, BMI, waist circumference, the percentages of alcohol consumption, the levels of diastolic blood pressure, TC, LDL-C, ApoB and the ratio of ApoA1 to ApoB between the two ethnic groups (*P* > 0.05 for all).

**Table 1:** Comparison of demographic, lifestyle characteristics and serum lipid levels between the Maonan and Han populations

Parameter	Maonan	Han	*t* (χ^2^)	*P*
Number	867	820		
Gender (Male/female)	329/538	306/514	0.071	0.790
Age (year)	57.71 ± 14.74	56.34 ± 15.63	0.672	0.513
Height (cm)	152.90 ± 11.90	153.99 ± 7.71	−1.425	0.154
Weight (kg)	52.50 ± 10.96	53.86 ± 9.20	−1.936	0.053
Body mass index (kg/m^2^)	22.21 ± 3.91	22.65 ± 3.11	−1.712	0.087
Waist circumference (cm)	75.86 ± 10.22	75.40 ± 7.89	0.730	0.466
Cigarette smoking [n(%)]				
Non-smoker	698(80.46)	617(75.25)	7.019	0.030
≤ 20 cigarettes per day	148(17.08)	181(22.04)		
> 20 cigarettes per day	21(2.46)	22(2.71)		
Alcohol consumption [n (%)]				
Non-drinker	707(81.60)	662(80.68)	2.351	0.309
≤ 25 g per day	80(9.20)	92(11.19)		
> 25 g per day	80(9.20)	66(8.13)		
Systolic blood pressure (mmHg)	135.73 ± 26.01	130.46 ± 19.72	3.353	0.001
Diastolic blood pressure (mmHg)	83.57 ± 13.61	82.52 ± 11.61	1.144	0.253
Pulse pressure (mmHg)	52.15 ± 17.91	47.95 ± 15.47	3.611	0.000
Glucose (mmol/L)	5.69 ± 2.03	5.97 ± 1.61	−2.045	0.041
Total cholesterol (mmol/L)	4.97 ± 1.08	4.97 ± 1.03	0.058	0.953
Triglyceride (mmol/L)	1.22(0.85)	1.1(0.88)	−2.632	0.009
HDL-C (mmol/L)	1.62 ± 0.40	1.69 ± 0.42	−2.286	0.023
LDL-C (mmol/L)	2.83 ± 0.81	2.88 ± 0.87	−0.878	0.380
Apolipoprotein (Apo) A1 (g/L)	1.39 ± 0.32	1.30 ± 0.25	4.219	0.000
ApoB (g/L)	0.87 ± 0.20	0.85 ± 0.20	1.603	0.109
ApoA1/ApoB	1.66 ± 0.61	1.61 ± 0.50	1.208	0.227

*HDL-C* high-density lipoprotein cholesterol, *LDL-C* low-density lipoprotein cholesterol. The value of triglyceride was presented as median (interquartile range), the difference between the two ethnic groups was determined by the Wilcoxon-Mann-Whitney test

### Results of electrophoresis and genotyping

**Fig. 1:**
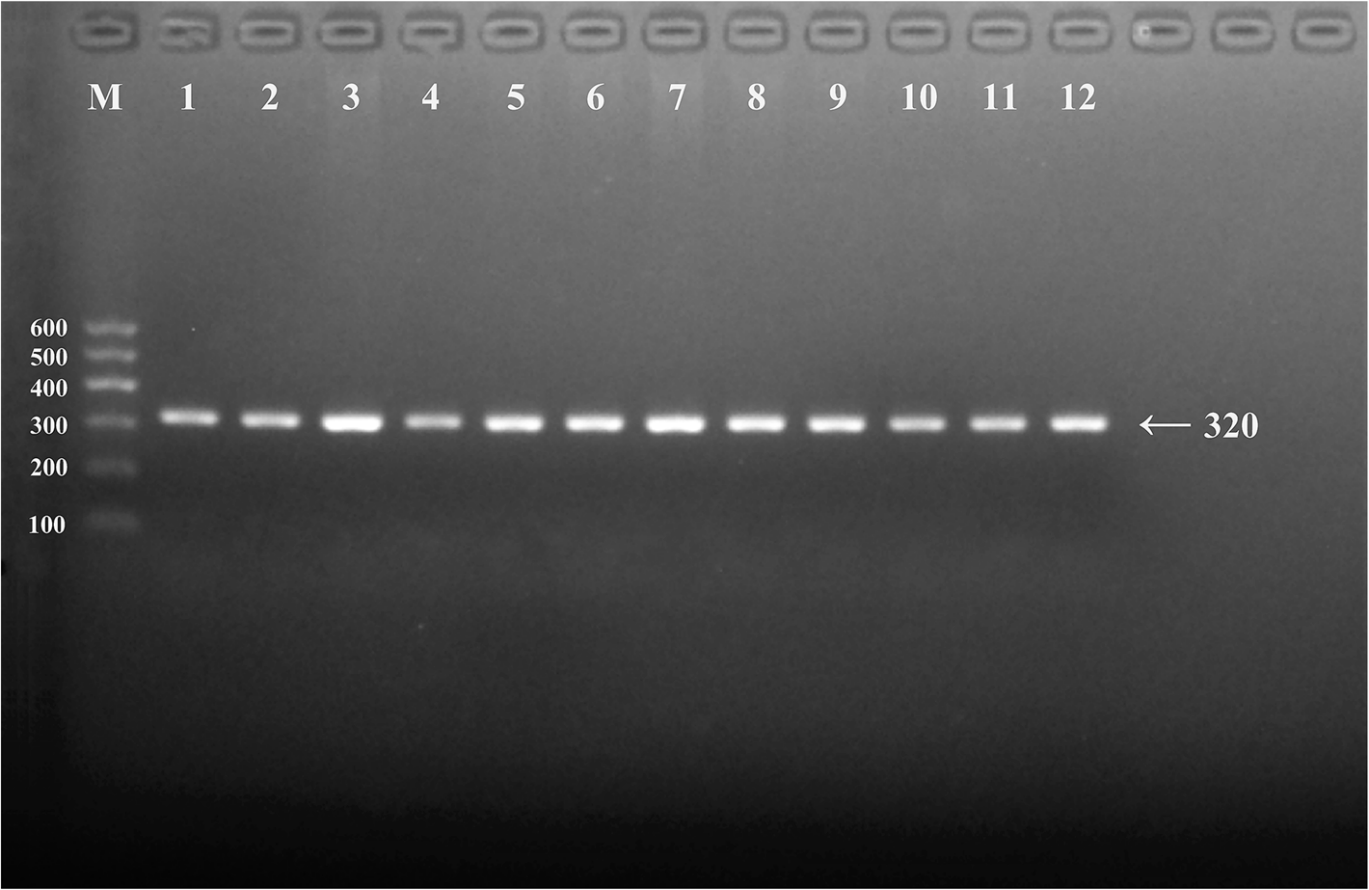
Electrophoresis of polymerase chain reaction products of the samples. Lane M is the 100 bp–600 bp marker ladder; Lanes 1-12 are samples, the 320 bp bands are the target genes

**Fig. 2:**
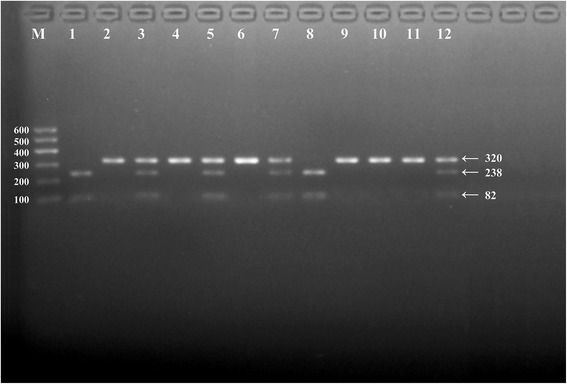
Genotyping of the *APOA1* rs964184 SNP. Lane M is the 100 bp-600 bp marker ladder. Lanes 2, 4, 6, 9, 10 and 11 are the CC genotype (320 bp); lanes 3, 5, 7 and 12 are the CG genotype (320-, 238- and 82- bp); lanes 1 and 8 are the GG genotype (238- and 82- bp)

**Fig. 3:**
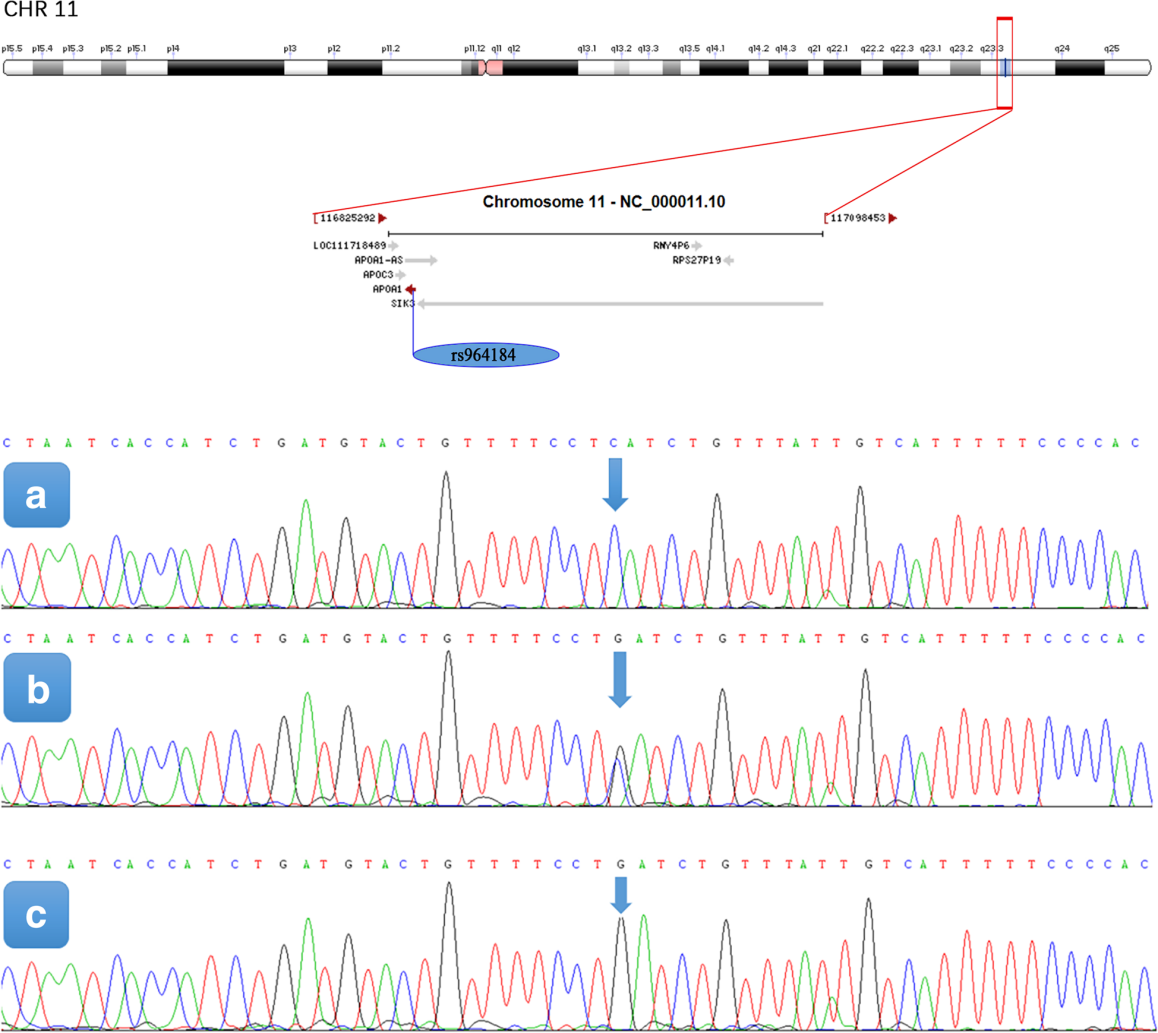
The position of the *APOA1* rs964184 SNP and a part of the nucleotides sequences of the *APOA1* rs964184 SNP. A, CC genotype; B, CG genotype; C, GG genotype

After the genomic DNA of the samples was amplified using PCR and visualized with 2% agarose gel electrophoresis, the products of 320 bp nucleotide sequences were observed in all samples (Fig. 1). The genotypes identified were termed according to the presence (G allele) or absence (C allele) of the enzyme restriction sites. Thus, the CC genotype is homozygous for the absence of the site (bands at 320 bp), the CG genotype is heterozygous for the presence and absence of the site (bands at 320-, 238- and 82- bp) and the GG genotype is homozygous for the presence of the site (bands at 238 bp and 82 bp; Fig. 2). The position of the *APOA1* rs964184 SNP and the CC, CG and GG genotypes confirmed by direct sequencing are shown in Fig. 3, respectively.

### Genotypic and allelic frequencies

The genotypic and allelic frequencies of the *APOA1* rs964184 SNP are shown in Table 2. The genotype distribution of the Maonan and Han populations was consistent with the Hardy-Weinberg equilibrium (HWE, *P* > 0.05) by the chi-square test of the goodness of fit. The frequencies of C and G alleles were 83.45 and 16.55% in Maonan, and 79.21 and 20.79% in Han populations (*P* < 0.05), respectively. The frequencies of CC, CG, and GG genotypes were 68.86, 29.18 and 1.96% in the Maonan population, and 63.78, 30.85 and 5.37% in the Han population (*P* < 0.05), respectively. No difference in the genotypic and allelic frequencies was found between males and females in each ethnic group (*P* > 0.05 for all).

**Table 2:** Comparison of the genotype and allele frequencies of the *APOA1* rs964184 SNP in the Maonan and Han populations [n (%)]

Group	n	Genotype			Allele		*P*_HWE_
		CC	CG	GG	C	G	
Maonan	867	597(68.86)	253(29.18)	17(1.96)	1447(83.45)	287(16.55)	0.097
Han	820	523(63.78)	253(30.85)	44(5.37)	1299(79.21)	341(20.79)	0.070
χ^*2*^		15.470			10.009		
*P*		0.000			0.002		
Maonan							
Male	329	231(70.21)	94(28.57)	4(1.22)	556(84.50)	102(15.50)	0.100
Female	538	366(68.03)	159(29.55)	13(2.42)	891(82.81)	185(17.19)	0.379
χ^*2*^		1.710			0.846		
*P*		0.425			0.358		
Han							
Male	306	206(67.32)	87(28.43)	13(4.58)	499(81.37)	113(18.63)	0.330
Female	514	317(61.67)	166(32.30)	31(6.03)	800(79.28)	228(20.72)	0.144
χ^*2*^		3.024			3.215		
*P*		0.221			0.073		

*HWE* Hardy-Weinberg equilibrium. The genotype distribution between the two groups was analyzed by the chi-square test. The HWE was analyzed by the chi-square test of the goodness of fit

### Genotypes and serum lipid levels

Tables 3 and 4 show the association between genotypes and serum lipid levels. Serum levels of HDL-C were different between the genotypes (*P* < 0.05) in Maonan but not in Han, the G allele carriers had lower serum HDL-C level than the G allele non-carriers. Subgroup analyses showed that the G allele carriers had lower serum HDL-C level in both Maonan males and females (*P* < 0.05). Serum TG levels in Han were different between the genotypes (*P <* 0.05), the G allele carriers had higher serum levels of TG than the G non-carriers. Subgroup analyses showed that there was no significant difference between Han males and females, but serum ApoA1 levels and the ApoA1/ApoB ratio were significant different in Han males, the G allele carriers had lower ApoA1 levels and lower ApoA1/ApoB ratio than the G allele non-carriers. No significant difference in the remaining lipid parameters was found between the genotypes in the both populations or in the males and females of the two ethnic groups.

**Table 3:** Comparison of the genotypes and serum lipid levels in the Maonan and Han populations

Group/Genotype	n	TC (mmol/L)	TG (mmol/L)	HDL-C (mmol/L)	LDL-C (mmol/L)	ApoA1 (g/L)	ApoB(g/L)	ApoA1/ ApoB
Maonan								
CC	597	5.01 ± 0.05	1.21(0.80)	1.65 ± 0.02	2.85 ± 0.04	1.40 ± 0.02	0.87 ± 0.01	1.68 ± 0.03
CG/GG	270	4.90 ± 0.08	1.34(1.04)	1.55 ± 0.03	2.78 ± 0.06	1.36 ± 0.02	0.87 ± 0.01	1.63 ± 0.04
*F*		1.138	−1.506	8.790	1.063	2.131	0.010	0.818
*P*		0.286	0.132	0.003	0.303	0.145	0.920	0.366
Han								
CC	523	4.97 ± 0.07	1.04(0.86)	1.71 ± 0.03	2.90 ± 0.06	1.31 ± 0.02	0.85 ± 0.01	1.63 ± 0.04
CG/GG	297	4.96 ± 0.10	1.23(1.16)	1.65 ± 0.04	2.84 ± 0.09	1.28 ± 0.02	0.86 ± 0.02	1.58 ± 0.05
*F*		0.002	−2.195	1.605	0.339	0.940	0.330	0.516
*P*		0.962	0.028	0.206	0.561	0.333	0.566	0.473

*TC* total cholesterol, *TG* triglyceride, *HDL-C* high-density lipoprotein cholesterol, *LDL-C* low-density lipoprotein cholesterol, *ApoA1* apolipoprotein A1, *ApoB* apolipoprotein B, *ApoA1/ApoB* the ratio of apolipoprotein A1 to apolipoprotein B. The value of TG was presented as median (interquartile range), the difference between the genotypes was determined by the Wilcoxon-Mann-Whitney test

**Table 4:** Comparison of the genotypes and serum lipid levels between males and females in the Maonan and Han populations

Ethnic/Genotype	n	TC (mmol/L)	TG (mmol/L)	HDL-C (mmol/L)	LDL-C (mmol/L)	ApoA1 (g/L)	ApoB (g/L)	ApoA1/ApoB
Maonan/Male								
CC	230	4.92 ± 0.07	1.26(0.87)	1.59 ± 0.03	2.76 ± 0.06	1.40 ± 0.04	0.87 ± 0.02	1.69 ± 0.06
CG/GG	99	4.85 ± 0.11	1.43(1.44)	1.45 ± 0.05	2.69 ± 0.09	1.33 ± 0.05	0.89 ± 0.02	1.54 ± 0.09
*F*		0.247	−0.837	6.996	0.317	1.452	0.412	1.784
*P*		0.620	0.403	0.009	0.574	0.230	0.521	0.183
Maonan/Female								
CC	366	5.06 ± 0.08	1.19(0.68)	1.69 ± 0.02	2.91 ± 0.05	1.41 ± 0.01	0.88 ± 0.01	1.67 ± 0.03
CG/GG	172	4.93 ± 0.11	1.31(0.88)	1.61 ± 0.04	2.84 ± 0.08	1.38 ± 0.02	0.86 ± 0.02	1.67 ± 0.05
*F*		1.001	−1.361	3.903	0.676	1.025	0.316	0.000
*P*		0.318	0.174	0.049	0.412	0.312	0.574	0.998
Han/Male								
CC	206	5.12 ± 0.11	1.11(0.95)	1.66 ± 0.04	2.92 ± 0.09	1.34 ± 0.03	0.89 ± 0.02	1.57 ± 0.05
CG/GG	100	4.98 ± 0.16	1.34(2.26)	1.52 ± 0.06	2.88 ± 0.13	1.23 ± 0.04	0.94 ± 0.03	1.38 ± 0.07
*F*		0.519	−1.140	3.669	0.077	5.286	1.359	5.272
*P*		0.473	0.254	0.058	0.782	0.023	0.246	0.024
Han/Female								
CC	317	4.87 ± 0.10	0.97(0.82)	1.74 ± 0.04	2.88 ± 0.08	1.30 ± 0.02	0.82 ± 0.02	1.67 ± 0.05
CG/GG	197	4.97 ± 0.14	1.15(0.83)	1.72 ± 0.05	2.83 ± 0.12	1.31 ± 0.03	0.82 ± 0.03	1.69 ± 0.07
*F*		0.328	−1.902	0.127	0.128	0.096	0.015	0.032
*P*		0.567	0.057	0.722	0.721	0.758	0.901	0.858

*TC* total cholesterol, *TG* triglyceride, *HDL-C* high-density lipoprotein cholesterol, *LDL-C* low-density lipoprotein cholesterol, *ApoA1* apolipoprotein A1, *ApoB* apolipoprotein B, *ApoA1/ApoB* the ratio of apolipoprotein A1 to apolipoprotein B. The value of triglyceride was presented as median (interquartile range), the difference among the genotypes was determined by the Wilcoxon-Mann-Whitney test

### Relative factors for serum lipid parameters

Multiple linear regression analysis showed that serum HDL-C and ApoA1 levels in the two populations were correlated with the genotypes of *APOA1* rs964184 SNP; likewise, serum HDL-C levels in Maonan and TG levels in Han were correlated with the genotypes of the *APOA1* rs964184 SNP (*P* < 0.05 for each; Table 5). When the correlation of serum lipid parameters and the genotypes was analyzed according to two genders, we found that serum HDL-C levels in Maonan males, ApoA1 levels, and the ApoA1/ApoB ratio in Han males, together with serum TG levels in Han females were all correlated with the genotypes (*P* < 0.05; Table 6). Serum lipid parameters were also associated with age, gender, BMI, waist circumference, systolic and diastolic blood pressure, pulse pressure, fasting blood glucose, cigarette smoking and alcohol consumption in both ethnic groups or in males and females (*P* < 0.05-0.001; Tables 5 and 6).

**Table 5:** Relationship between serum lipid parameters and relative factors in the Maonan and Han populations

Lipid	Risk factor	B	Std. error	Beta	*t*	*P*
Maonan and Han						
TC	Age	0.010	0.003	0.146	3.679	0.000
	Height	−0.014	0.007	−0.124	−2.033	0.042
	Waist circumference	0.024	0.006	0.204	3.911	0.000
TG	Alcohol consumption	0.006	0.002	0.114	3.063	0.002
	Height	−0.025	0.010	− 0.149	−2.506	0.012
	Weight	0.047	0.018	0.300	2.581	0.010
	Waist circumference	0.039	0.009	0.223	4.396	0.000
HDL-C	Gender	0.161	0.039	0.191	4.162	0.000
	Alcohol consumption	0.002	0.000	0.183	4.935	0.000
	Waist circumference	−0.009	0.002	− 0.212	−4.198	0.000
	Pulse pressure	−0.002	0.001	−0.074	−2.043	0.041
	Ethnic group	0.070	0.028	0.082	2.497	0.013
	Genotype	−0.051	0.014	−0.115	−3.591	0.000
LDL-C	Age	0.009	0.002	0.168	4.305	0.000
	Alcohol consumption	−0.003	0.001	−0.120	− 3.163	0.001
	Waist circumference	0.019	0.005	0.211	4.135	0.000
ApoA1	Gender	0.089	0.030	0.143	3.025	0.003
	Cigarette smoking	0.004	0.002	0.103	2.558	0.011
	Alcohol consumption	0.002	0.000	0.174	4.569	0.000
	Waist circumference	−0.004	0.002	−0.108	−2.083	0.038
	Ethnic group	−0.091	0.022	−0.141	−4.204	0.000
	Genotype	−0.023	0.011	−0.069	−2.103	0.036
ApoB	Age	0.002	0.000	0.148	3.878	0.000
	Height	−0.003	0.001	−0.130	−2.234	0.026
	Waist circumference	0.007	0.001	0.330	6.614	0.000
ApoA1/ApoB	Gender	0.177	0.054	0.148	3.273	0.001
	Age	−0.004	0.001	− 0.114	−2.977	0.003
	Cigarette smoking	0.008	0.003	0.103	2.661	0.008
	Alcohol consumption	0.003	0.001	0.176	4.801	0.000
	Waist circumference	−0.018	0.003	−0.288	−5.773	0.000
	Ethnic group	−0.086	0.040	−0.070	−2.162	0.031
Maonan						
TC	Age	0.009	0.004	0.127	2.621	0.009
	Gender	0.276	0.129	0.124	2.138	0.033
	Waist circumference	0.024	0.007	0.218	3.389	0.001
TG	Height	−0.023	0.011	−0.134	−2.006	0.045
	Weight	0.045	0.021	0.287	2.119	0.035
	Alcohol consumption	0.008	0.002	0.170	3.574	0.000
	Waist circumference	0.038	0.011	0.217	3.471	0.001
HDL-C	Gender	0.166	0.046	0.200	3.623	0.000
	Alcohol consumption	0.002	0.001	0.209	4.470	0.000
	Waist circumference	−0.010	0.003	−0.235	−3.825	0.000
	Pulse pressure	− 0.002	0.001	− 0.096	−2.129	0.034
	Genotype	−0.055	0.017	−0.124	−3.179	0.002
LDL-C	Age	0.008	0.003	0.148	3.161	0.002
	Alcohol consumption	−0.004	0.001	−0.183	− 3.858	0.000
	Waist circumference	0.021	0.005	0.247	3.969	0.000
ApoA1	Gender	0.092	0.039	0.138	2.392	0.017
	Alcohol consumption	0.001	0.000	0.149	3.051	0.002
	Waist circumference	−0.005	0.002	−0.137	−2.131	0.034
ApoB	Age	0.002	0.001	0.159	3.470	0.001
	Waist circumference	0.008	0.001	0.376	6.176	0.000
ApoA1/ApoB	Age	−0.005	0.002	−0.131	−2.852	0.005
	Alcohol consumption	0.003	0.001	0.183	3.955	0.000
	Waist circumference	−0.020	0.004	−0.325	−5.336	0.000
Han						
TC	Age	0.010	0.005	0.147	2.133	0.034
	Diastolic blood pressure	0.014	0.005	0.162	2.670	0.008
TG	Cigarette smoking	0.035	0.012	0.202	3.056	0.002
	Waist circumference	0.043	0.016	0.245	2.696	0.007
	Diastolic blood pressure	0.015	0.007	0.129	2.207	0.028
	Genotype	0.248	0.083	0.164	2.993	0.003
HDL-C	Gender	0.166	0.074	0.193	2.247	0.025
	Alcohol consumption	0.002	0.001	0.152	2.426	0.016
LDL-C	Age	0.010	0.004	0.170	2.457	0.015
	Cigarette smoking	−0.018	0.008	−0.163	−2.384	0.018
ApoA1	Gender	0.097	0.045	0.187	2.172	0.031
	Alcohol consumption	0.002	0.001	0.263	4.190	0.000
	Cigarette smoking	0.006	0.002	0.173	2.497	0.013
ApoB	Gender	−0.099	0.034	−0.236	−2.881	0.004
	Waist circumference	0.006	0.002	0.226	2.476	0.014
ApoA1 /ApoB	Gender	0.358	0.087	0.343	4.123	0.000
	Cigarette smoking	0.011	0.004	0.171	2.550	0.011
	Alcohol consumption	0.003	0.001	0.161	2.648	0.009

*TC* total cholesterol, *TG* triglyceride, *HDL-C* high-density lipoprotein cholesterol, *LDL-C* low-density lipoprotein cholesterol, *ApoA1* apolipoprotein A1, *ApoB* apolipoprotein B, *ApoA1/ApoB* the ratio of apolipoprotein A1 to apolipoprotein B, *B* unstandardized coefficient, *Beta* standardized coefficient

**Table 6:** Relationship between serum lipid parameters and relative factors in the males and females of the both ethnic groups

Lipid	Risk factor	B	Std. error	Beta	*t*	*P*
Maonan/ male						
TG	Alcohol consumption	0.010	0.004	0.188	2.605	0.010
HDL-C	Alcohol consumption	0.003	0.001	0.323	4.928	0.000
	Waist circumference	−0.018	0.005	−0.480	−3.884	0.000
	Genotype	−0.070	0.027	−0.155	−2.588	0.010
LDL-C	Alcohol consumption	−0.004	0.001	−0.274	−3.851	0.000
ApoA1	Alcohol consumption	0.002	0.001	0.175	2.420	0.016
	Waist circumference	−0.014	0.005	−0.344	−2.503	0.013
ApoB	Weight	0.008	0.003	0.432	2.449	0.015
ApoA1/ApoB	Age	−0.009	0.004	−0.174	−2.256	0.025
	Alcohol consumption	0.003	0.001	0.212	3.080	0.002
	Waist circumference	−0.022	0.009	−0.310	−2.391	0.018
Maonan/ female						
TC	Age	0.011	0.005	0.141	2.335	0.020
	Waist circumference	0.029	0.010	0.209	2.947	0.003
TG	Waist circumference	0.026	0.006	0.308	4.530	0.000
LDL-C	Age	0.009	0.003	0.157	2.688	0.008
	Waist circumference	0.029	0.007	0.297	4.336	0.000
ApoB	Age	0.002	0.001	0.164	2.910	0.004
	Waist circumference	0.010	0.002	0.406	6.135	0.000
ApoA1 /ApoB	Waist circumference	−0.018	0.004	−0.308	−4.507	0.000
	Pulse pressure	−0.004	0.002	−0.131	−2.336	0.020
Han/ male						
TG	Cigarette smoking	0.035	0.014	0.239	2.581	0.011
HDL-C	Alcohol consumption	0.002	0.001	0.194	2.099	0.038
	Diastolic blood pressure	0.008	0.004	0.246	2.253	0.026
LDL-C	Cigarette smoking	−0.018	0.008	−0.234	−2.381	0.019
ApoA1	Cigarette smoking	0.006	0.002	0.245	2.696	0.008
	Alcohol consumption	0.002	0.001	0.376	4.258	0.000
	Genotype	−0.054	0.026	−0.185	−2.074	0.041
ApoA1 /ApoB	Cigarette smoking	0.012	0.004	0.271	3.012	0.003
	Alcohol consumption	0.003	0.001	0.258	2.952	0.004
	Genotype	−0.091	0.045	−0.180	−2.040	0.044
Han/ female						
TC	Age	0.091	0.007	0.267	2.898	0.004
TG	Diastolic blood pressure	0.021	0.009	0.183	2.308	0.022
	Genotype	0.311	0.098	0.229	3.163	0.002
LDL-C	Age	0.023	0.005	0.380	4.254	0.000
	Height	0.176	0.073	1.156	2.426	0.016
	Weight	−0.270	0.109	−2.179	−2.487	0.014
	Body mass index	0.616	0.246	2.045	2.506	0.013
ApoB	Body mass index	0.112	0.055	1.698	2.025	0.044

*TC* total cholesterol, *TG* triglyceride, *HDL-C* high-density lipoprotein cholesterol, *LDL-C* low-density lipoprotein cholesterol, *ApoA1* apolipoprotein A1, *ApoB* apolipoprotein B, *ApoA1/ApoB* the ratio of apolipoprotein A1 to apolipoprotein B, *B* unstandardized coefficient, *Beta* standardized coefficient. The correlation among serum lipid parameters and the genotypes and several environmental factors was determined by multivariable linear regression analyses with stepwise modeling

## Discussion

The results of our study showed that the serum lipid profiles were different between the Maonan and Han populations. Serum TG and ApoA1 levels were higher in Maonan than in Han (*P* < 0.05-0.001), whereas the levels of serum HDL-C were lower in Maonan than in Han (*P* < 0.001). There was no significant difference in the levels of serum TC, LDL-C, ApoB and the ratio of ApoA1 to ApoB between the two ethnic groups (*P* > 0.05 for all). It was widely realized that dyslipidemia as a serious risk factor for CAD is caused by various elements, mainly including genetic and environmental factors and their interaction [24, 25]. Maonan nationality belongs to a mountain ethnic minority and is mainly occupied with cereal and miscellaneous grain crops. The history of Maonan can retrospect to the eleventh century. According to the statistics in 2000, the numbers of Maonan population were 107,166, mainly engaged in agriculture and were good at raising beef cattle and prepare the bamboo hat. The main food for them was rice, along with corn, sorghum, millet, sweet potatoes and pumpkin which are also important complements. Thus, they enjoyed a very special lifestyle and dietary habits compared with the other nationalities. Maonan people were fond of spicy and acid food. Parents usually were in charge of their children’s marriages. Maonan stays endogamy; intermarriage with Han or Zhuang people is seldom happened. Therefore, it is considered that the hereditary characteristics and genotypes of certain lipid metabolism-related genes in this population might be different from those in the Han population.

According to the International HapMap Project’s data-base, the frequencies of G allele and CG, GG genotypes were 20.73, 31.71 and 4.88% in Chinese Han in Beijing; 12.05, 22.32 and 0.89% in European; and 33.14, 52.33 and 6.98% in Japanese, respectively. While the genotypic and allelic frequencies of the *APOA1* rs964184 SNP have not been reported previously in different ethnic groups. In the present study, we firstly showed that the G allele frequency of the *APOA1* rs964184 SNP was lower in Maonan than in Han populations (16.55% vs. 20.79%, *P* < 0.01). The distribution of the genotypes was also significantly different between the two ethnic groups (*P* < 0.001), the frequencies of CG and GG genotypes were lower in Maonan than in Han groups, respectively. No significant difference was observed in the genotypic and allelic frequencies between males and females in the two ethnic groups. These results indicate that the prevalence of *APOA1* rs964184 SNP may have racial/ethnic specificity.

To the best of our knowledge, the potential association of the *APOA1* rs964184 SNP and serum lipid levels has not been previously reported in different racial/ethnic groups. A previous associated study indicated that the *APOA1* encodes a protein, ApoA1, in human beings [12]. ApoA1 could be a better biomarker of cardiovascular risk [10], and it was associated with HDL-C, in another word, it was the most abundant component of HDL-C [14], which played an essential role in formation, maturation and metabolism of HDL-C [15]. Another study about Brazilian people showed that this protein influenced lipid levels and may be risk factors for CVD in the Brazilian elderly [26]. Recently, another GWAS has also identified that genetic variant of the *APOA1* rs964184 SNP was associated with serum TG levels mainly, and with TC, HDL-C, LDL-C, secondly [16]. In the current study, we firstly showed that the G allele carriers in Maonan had lower serum HDL-C levels than the G allele non-carriers; the G allele carriers in Han had lower serum TG levels than the G allele non-carriers. Subgroup analyses showed that the G allele carriers in both Maonan males and females had lower serum HDL-C levels, and the G allele carriers in Han males had lower serum ApoA1 levels and the ApoA1/ApoB ratio than the G allele non-carriers. These findings suggest that there may be an ethnic- and gender-specific association of the *APOA1* rs964184 SNP and serum lipid levels.

Previous studies showed that environmental factors such as dietary patterns, lifestyle and physical inactivity are strongly related with serum lipid levels [27]. In the present study, multivariate linear regression analysis also showed that serum lipid parameters were correlated to age, gender, waist circumference, BMI, blood pressure, blood glucose, alcohol consumption, and cigarette smoking in both ethnic groups. These findings suggest that the environmental factors also play an important role in determining or altering serum lipid levels in our study populations. The dietary habits are different between the Maonan and Han populations. Rice is the Maonan people’s staple food supplemented with corn, sweet potato and other grains. Maonan people are fond of very strong flavor food, such as eating spicy and acid food with lots of oil and salt. This preference of high in carbohydrates may be related to the higher blood glucose levels, weight, BMI and waist circumference in Maonan than in Han people. In the meantime, rich oil and salt can give rise to higher blood pressure, serum TC, LDL-C and ApoB levels in Maonan than in Han people. Many previous studies proved that diet alone could account for the variability on serum lipid levels [28, 29].

In addition, we also observed that the percentage of cigarette smoking was lower in Maonan than in Han (*P* < 0.05). In multiple linear regression analysis, we could find that alcohol consumption and cigarette smoking may influence serum TC, TG, HDL-C, ApoA1 levels and the ApoA1/ApoB ratio (*P* < 0.05). Several case-control and cohort studies have described a J- or U-shaped association between alcohol intake and atherogenesis [30]. A moderate intake of alcohol when taken on a regular amount has been showed to protect against CAD death, which has been attributed to the alterations in serum HDL-C, TG and ApoA1 levels [31]. However, alcohol consumption was also associated with worse hematological values of TC and LDL-C levels. Another research indicated that the effects of alcohol consumption on LDL-C appear to vary by specific patient types or patterns of alcohol intake, and gender as well as genetic variants [32]. In terms of cigarette smoking, one of previous study described that cigarette smoking could be a primary factor in potential changes in lipid profile, which may give rise to the onset of atherosclerosis and CAD in the future [33]. Another study showed that there was difference between two cigarette smoking habits: the length period of smoking and a number of cigarettes smoked daily, and made a conclusion that more reflection to the status of lipids has the bigger number of smoked cigarettes daily than the length of the period of cigarette smoking [34]. Therefore, the results of exposure to different lifestyle and environmental factors probably further modify the association of genetic variations and serum lipid levels in our study populations.

### Limitations

There are several potential limitations in our study. Firstly, we were not able to alleviate the effect of diet and some environmental factors during the statistical analysis. Secondly, we could not completely exclude asymptomatic disorders, atherosclerosis, for instance, which could create a potentially significant bias due to poor field study condition. Thirdly, there are still many unmeasured environmental and genetic factors which should be considered, although we have observed significant association of the *APOA1* rs964184 SNP and serum lipid levels. In addition, the interactions of gene-gene, gene-environment, and environment-environment on serum lipid levels are remained to be confirmed.

## Conclusions

The present study showed that the genotypic and allelic frequencies of the *APOA1* rs964184 SNP were significantly different between the Maonan and Han populations. The associations of the *APOA1* rs964184 SNP and serum lipid levels were also significantly different between the two ethnic groups and between males and females in the Maonan population. There may be a racial/ethnic- and/or gender-specific association of the *APOA1* rs964184 SNP and serum lipid levels.

## Abbreviations

ANCOVA: Analysis of covariance
Apo: Apolipoprotein
APOA1: Apolipoprotein A1
BMI: Body mass index
CAD: Coronary artery disease
CVD: Cardiovascular disease
GWAS: Genome-wide association study
HDL-C: High-density lipoprotein cholesterol
HWE: Hardy-Weinberg equilibrium
LDL-C: Low-density lipoprotein cholesterol
PCR: Polymerase chain reaction
RFLP: Restriction fragment length polymorphism
SNP: Single nucleotide polymorphism
TC: Total cholesterol
TG: Triglyceride
